# Whole-Genome Sequencing of *Lactobacillus* Species from Two Commercial Probiotic Products

**DOI:** 10.1128/genomeA.01279-17

**Published:** 2017-11-09

**Authors:** Anna Colavecchio, Victoria Leo, Sophia Zaccheo, Julie Jeukens, Jean-Guillaume Emond-Rheault, Jérémie Hamel, Irena Kukavica-Ibrulj, Roger C. Levesque, Lawrence Goodridge

**Affiliations:** a Department of Food Science and Agricultural Chemistry, Food Safety and Quality Program, McGill University, Québec, Canada; b Institut de Biologie Intégrative et des Systèmes (IBIS), Université Laval, Québec, Québec, Canada

## Abstract

Eight *Lactobacillus* strains, each intrinsically resistant to an antibiotic, were isolated from two commercial probiotic products. Whole-genome sequencing identified two efflux transporters, a multidrug and extrusion protein (MATE) efflux transporter, and LmrCD, which may contribute to their intrinsic antibiotic resistance and may therefore facilitate their survival in the intestinal microbiota following antibiotic therapy.

## GENOME ANNOUNCEMENT

Probiotics are live microorganisms that confer many health-promoting effects when consumed in adequate amounts. Many probiotic bacteria are naturally resistant to various antibiotics, with resistance conferred by intrinsic mechanisms, such as multidrug efflux transporters (1, 2).

Eight *Lactobacillus* strains were isolated from two commercial probiotic products, and each was found to be resistant to an antibiotic of clinical importance. We performed whole-genome sequencing (WGS) on these isolates to determine the nature of their resistance.

*Lactobacillus* strains were isolated by performing serial dilutions of two commercial probiotic products and plating onto DeMan-Rogosa-Sharpe (MRS) medium (Sigma-Aldrich, Ontario, Canada) supplemented with gentamicin (5 µg/ml), streptomycin (10 µg/ml), kanamycin (30 µg/ml), aztreonam (30 µg/ml), and ciprofloxacin (10 µg/ml) and incubated anaerobically for 48 h at 35°C (3). WGS was performed at the EcoGenomics analysis platform (IBIS, Université Laval, Québec, Canada) on an Illumina MiSeq instrument using 300-bp paired-end libraries with 40× coverage. The raw reads were assembled using the A5 pipeline (4). A WGS BLAST search (5) identified the genus and species of the isolates, which corresponded with results from the Vitek microbial identification system (bioMérieux, Quebec, Canada). Rapid Annotation of microbial genomes using Subsystems Technology (RAST) was used for annotation (6), while RAST and CARD (7) identified antibiotic resistance and heavy-metal resistance genes.

The eight *Lactobacillus* isolates were identified as *Lactobacillus rhamnosus* B1 (2,908,459 bp, 46.8% G+C content), *Lactobacillus paracasei* B2 (3,013,831 bp, 46.3% G+C content), *Lactobacillus paracasei* B3 (3,015,056 bp, 46.3% G+C content), *Lactobacillus casei* P1 (2,967,632 bp, 46.8% G+C content), *Lactobacillus acidophilus* P2 (2,046,837 bp, 35.7% G+C content), *Lactobacillus casei* P3 (2,971,889 bp, 46.8% G+C content), *Lactobacillus rhamnosus* P4 (2,952,578 bp, 46.7% G+C content), and *Lactobacillus casei* P5 (2,987,029 bp, 46.9% G+C content). WGS revealed that the *Lactobacillus* isolates carry two types of multidrug transport proteins from two families, a multidrug and extrusion protein (MATE) family efflux transporter and an ATP-binding cassette (ABC) superfamily protein called LmrCD. These families of transporters play essential roles in the intrinsic and acquired resistance to antibiotics in numerous species of bacteria (8, 9). The MATE efflux transporter has been reported to confer resistance to ciprofloxacin, gentamicin, streptomycin, and kanamycin. Moreover, it is located on the chromosomes of the *Lactobacillus* isolates and not flanked by mobile genetic elements; hence, we hypothesize that it may play a crucial role in the intrinsic resistance to the four antibiotics tested (9). WGS also revealed that each genome confers resistance to fluoroquinolones via the *gyrA* and *parC* genes and β-lactamase class A and C resistance. Furthermore, all the genomes carry the copper oxidase precursor protein CueO, cytoplasmic copper homeostasis protein CutC, and a cobalt-cadmium-zinc and mercury-transporting ATPase resistance gene. These heavy-metal resistance genes have been reported in *Lactobacillus* spp. to sequester heavy metals present in the intestinal microbiota protecting the host (10).

To summarize, WGS suggests that two efflux transporters, a MATE transporter and LmrCD, may contribute to the intrinsic antibiotic resistance of eight *Lactobacillus* isolates. The intrinsic resistance to antibiotics of clinical importance may help sustain these probiotic bacteria in the intestinal microbiota during and following antibiotic therapy, providing health benefits to the host.

### Accession number(s).

The complete genome sequences have been deposited in GenBank as follows: *Lactobacillus rhamnosus* B1, accession no. NXEU00000000; *Lactobacillus paracasei* B2, accession no. NXET00000000; *Lactobacillus paracasei* B3, accession no. NXES00000000; *Lactobacillus casei* P1, accession no. NXEZ00000000; *Lactobacillus acidophilus* P2, accession no. NXEY00000000; *Lactobacillus casei* P3, accession no. NXEX00000000; *Lactobacillus rhamnosus* P4, accession no. NXEW00000000; and *Lactobacillus casei* P5, accession no. NXEV00000000.
